# Cost-effectiveness of an HPV self-collection campaign in Uganda: comparing models for delivery of cervical cancer screening in a low-income setting

**DOI:** 10.1093/heapol/czw182

**Published:** 2017-02-20

**Authors:** Nicole G Campos, Vivien Tsu, Jose Jeronimo, Denise Njama-Meya, Mercy Mvundura, Jane J Kim

**Affiliations:** 1 Center for Health Decision Science, Harvard T.H. Chan School of Public Health, 718 Huntington Avenue, Boston, MA, USA; 2 PATH, Reproductive Health Global Program, P.O. Box 900922, Seattle, WA, USA; 3 PATH Uganda, P.O. Box 7404, Kampala, Uganda; 4 PATH, Devices and Tools Program, P.O. Box 900922, Seattle, WA, USA

**Keywords:** Cancer, cervical screening, cost-effectiveness analysis, decision making, women’s health

## Abstract

With the availability of a low-cost HPV DNA test that can be administered by either a healthcare provider or a woman herself, programme planners require information on the costs and cost-effectiveness of implementing cervical cancer screening programmes in low-resource settings under different models of healthcare delivery. Using data from the START-UP demonstration project and a micro-costing approach, we estimated the health and economic impact of once-in-a-lifetime HPV self-collection campaign relative to clinic-based provider-collection of HPV specimens in Uganda. We used an individual-based Monte Carlo simulation model of the natural history of HPV and cervical cancer to estimate lifetime health and economic outcomes associated with screening with HPV DNA testing once in a lifetime (clinic-based provider-collection vs a self-collection campaign). Test performance and cost data were obtained from the START-UP demonstration project using a micro-costing approach. Model outcomes included lifetime risk of cervical cancer, total lifetime costs (in 2011 international dollars [I$]), and life expectancy. Cost-effectiveness ratios were expressed using incremental cost-effectiveness ratios (ICERs). When both strategies achieved 75% population coverage, ICERs were below Uganda’s per capita GDP (self-collection: I$80 per year of life saved [YLS]; provider-collection: I$120 per YLS). When the self-collection campaign achieved coverage gains of 15–20%, it was more effective than provider-collection, and had a lower ICER unless coverage with both strategies was 50% or less. Findings were sensitive to cryotherapy compliance among screen-positive women and relative HPV test performance. The primary limitation of this analysis is that self-collection costs are based on a hypothetical campaign but are based on unit costs from Uganda. Once-in-a-lifetime screening with HPV self-collection may be very cost-effective and reduce cervical cancer risk by > 20% if coverage is high. Demonstration projects will be needed to confirm the validity of our logistical, costing and compliance assumptions.

Key Messages

- As governments and donors consider how to harness scarce global health resources to achieve the greatest health impact, information is needed on the costs and cost-effectiveness of implementing cervical cancer screening programmes in settings with limited infrastructure, under different models of healthcare delivery.
- Using data from the START-UP demonstration project and a micro-costing approach, we estimated the health and economic impact of a once-in-a-lifetime HPV self-collection campaign relative to clinic-based provider-collection of HPV specimens in Uganda.
- We found that an HPV self-collection campaign would be an effective and very cost-effective alternative to clinic-based provider-collection in Uganda, particularly at high coverage levels (i.e. 75% and above) and when self-collection is associated with greater coverage of screening-eligible women than provider-collection.
- The chronic shortage of healthcare workers in low-resource settings will likely be a barrier to scale-up of clinic-based screening by a trained provider. By shifting the task of screening to community health workers and women, a model of care involving self-collection of HPV specimens has the potential to circumvent provider shortages and concentrate limited provider time on treatment of screen-positive women. Given the importance of screening coverage and the potential for increased uptake with self-collection, programmes may achieve greater health benefits by tailoring programmes to different segments of the population than by offering a one-size-fits-all approach.

## Introduction

Cervical cancer is caused by persistent infection with one or more oncogenic human papillomavirus (HPV) types. Despite the potential for prevention through organized screening programmes that detect and treat precancerous lesions, cervical cancer is a leading cause of cancer death among women worldwide (Ferlay *et al.* 2013). Routine screening with Pap smear testing has reduced the burden of cervical cancer in high-income countries (Kitchener *et al.* 2006), but the implementation of Pap-based screening programmes has not been feasible in low-resource settings due to a lack of healthcare delivery infrastructure and limited health budgets. Nearly 90% of cervical cancer deaths occur in the developing world, with the greatest toll in Eastern Africa (Ferlay *et al.* 2013). In Uganda, where current screening efforts are limited to opportunistic screening with Pap testing or visual inspection with acetic acid (VIA), there were an estimated 3900 new cases and 2300 deaths in 2012 (Ferlay *et al.* 2013).

Despite the challenges of implementing and scaling cervical cancer screening programmes in low-resource settings, several clinical and economic studies have suggested that a screen-and-treat approach using VIA or HPV DNA testing can be feasible, beneficial, and cost-effective (Sankaranarayanan *et al.* 2009; Shastri *et al.* 2014; Campos *et al.*2015a,b). The World Health Organization (WHO) recommends primary screening with HPV testing when resources are available due to its high sensitivity to detect precancer (World Health Organization 2013). Recent advances in technology have addressed logistical challenges to implementing HPV testing in low-resource settings, including constraints related to test administration, laboratory processing and cost. HPV specimens can be collected by a healthcare provider (provider-collection) or by the woman herself (self-collection), potentially reducing barriers to screening and increasing uptake by eliminating the need for a pelvic exam by a provider. A public-private collaboration has led to the development of careHPV (QIAGEN, Gaithersburg, MD), a lower-cost HPV DNA test that can be processed in clinics or laboratories with limited infrastructure. The performance of careHPV has been validated in demonstration projects, which found the sensitivity of careHPV from self-collected specimens to be 77% compared with 89% for provider-collected cervical specimens in Uganda (Qiao *et al.* 2008; Jeronimo *et al.* 2014). The decrement in test sensitivity associated with self-collection of vaginal samples poses a trade-off between the potential for increased uptake with self-sampling vs the enhanced lesion detection associated with provider-collection of cervical samples (Arbyn *et al.* 2014).

Although roll-out of an HPV vaccination programme for young adolescent girls in Uganda was initiated in 2015 with assistance from Gavi, the Vaccine Alliance (2014), the vaccine will not begin to reduce the burden of cervical cancer for 20 years. In the interim, two to three generations of women beyond the age of vaccination will face a high lifetime risk of cervical cancer. As governments and donors consider how to harness scarce global health resources to achieve the greatest health impact, information is needed on the costs and cost-effectiveness of implementing cervical cancer screening programmes in settings with limited infrastructure, under different models of healthcare delivery. Our objective was to estimate the health and economic impact of a once-in-a-lifetime HPV self-collection screening campaign relative to clinic-based provider-collection of HPV specimens in Uganda.

## Materials and methods

### Analytic overview

We used an existing individual-based Monte Carlo simulation model of the natural history of HPV and cervical cancer to estimate lifetime health and economic outcomes associated with screening with HPV DNA testing once in a woman’s lifetime, with specimens collected through either clinic-based provider-collection or a one-time self-collection campaign. The model was calibrated to epidemiologic data from Uganda (Campos *et al.* 2015b). Test performance data for both strategies and cost data for the provider-collection strategy were obtained from the START-UP demonstration project in Kampala, Uganda (Jeronimo *et al.* 2014); cost data for the self-collection campaign were informed by clinical experts and providers working in Uganda and were estimated using a micro-costing approach. Model outcomes included lifetime risk of cervical cancer, total lifetime costs (in 2011 international dollars [I$]) and life expectancy. Cost-effectiveness outcomes were expressed using incremental cost-effectiveness ratios (ICERs), defined as the additional cost of a particular strategy divided by its additional health benefit, compared with the next most costly strategy after eliminating strategies that are dominated (defined as more costly and less effective, or having higher ICERs than more effective options). Although there is no universal criterion that defines a threshold cost-effectiveness ratio, we considered the heuristic that an intervention with an ICER less than Uganda’s per capita gross domestic product (GDP) would be ‘very cost-effective’ and less than three times per capita GDP would be ‘cost-effective’ (World Health Organization 2001). Consistent with guidelines for cost-effectiveness analysis, we adopted a societal perspective, including costs irrespective of the payer, and discounted future costs and life-years at a rate of 3% per year to account for time preferences (Gold 1996; Tan-Torres Edejer 2003; Jamison *et al.* 2006).

### Mathematical simulation model

The natural history model of cervical carcinogenesis in an individual woman is represented as a sequence of monthly transitions between mutually exclusive health states, including type-specific HPV infection status, grade of precancer [i.e. cervical intraepithelial neoplasia (CIN) grade 2 or 3], and stage of invasive cancer (Campos *et al.* 2014, 2015a,b). Transition probabilities may vary by age, HPV type, duration of infection or precancerous lesion status, and prior HPV infection. Cancer detection can occur through symptoms or via screening. Each month, death can occur from non-cervical causes or from cervical cancer after its onset. The model tracks disease progression and regression, clinical events and economic outcomes over the lifetime of each individual woman, which are then aggregated for analysis.

Details of the model parameterization process, including calibration have been previously published in Campos *et al.* (2014) and Campos (2015a, b). Briefly, we estimated baseline ‘prior’ input parameter values and set plausible ranges for natural history transitions using epidemiologic data (Munoz *et al.* 2004; Kim *et al.* 2007; Sankaranarayanan *et al.* 2010; Herrero *et al.* 2011; Surveillance Epidemiology and End Results 2011). Repeated model simulations in the absence of any intervention were conducted, in which a single random value for each uncertain parameter was selected from the plausible range, creating a unique natural history input parameter set. We then computed a goodness-of-fit score by summing the log-likelihood of model-projected outcomes for each unique parameter set to represent the quality of fit to epidemiologic data from Uganda (i.e. calibration targets). We selected the top 50 input parameter sets that produced a good fit to the epidemiologic data to use in analyses as a form of probabilistic sensitivity analysis (Goldhaber-Fiebert *et al.* 2007; Kim *et al.* 2007; Campos *et al.* 2014) (Supplementary Data S1). We report results as the mean across these top 50 parameter sets; ICERs are reported as the ratio of the mean costs divided by the mean effects of one strategy vs another across sets (Stinnett and Paltiel 1997).

### Strategies

**Figure 1.**
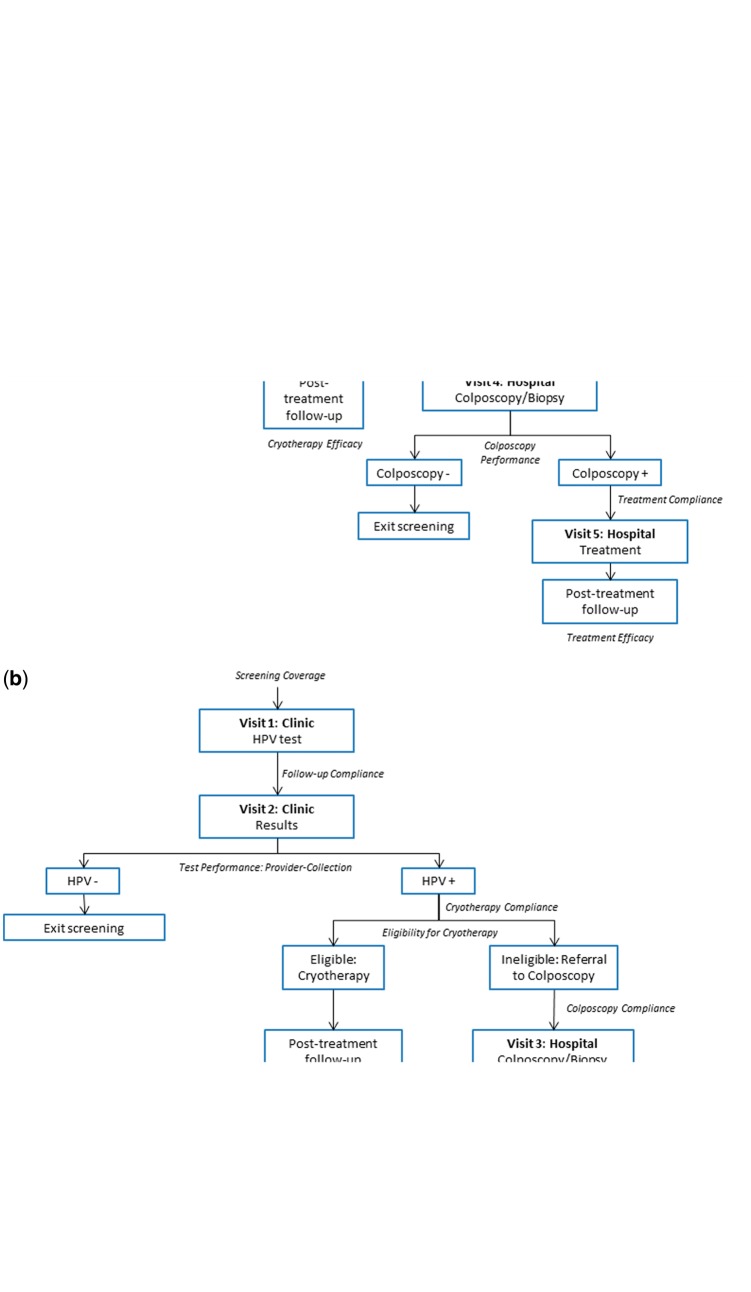
Pathways of health care delivery: Self-collection campaign vs clinic-based provider-collection. The diagrams indicate the flow of screening-eligible women through each point of contact in the screening and treatment process for (a) a onetime self-collection campaign and (b) clinic-based provider-collection.

We compared a one-time HPV self-collection campaign to clinic-based provider collection for women aged 30–49 years, as the WHO recommends prioritizing this age group for screening and a previous analysis found screening in this range to be very cost-effective in Uganda (World Health Organization 2013; Campos *et al.* 2015b). In collaboration with clinical experts and providers working in Uganda, we designed a pathway of care for the self-collection campaign that capitalized on existing health service delivery infrastructure (Figure 1a). We assumed the campaign, facilitated by community health workers (CHWs), would consist of monthly group sessions in a prototypical primary health facility catchment area held over a period of 6 months. Following mobilization efforts, each self-collection session would be held in a local school, church or meeting hall, during which a health provider and CHWs would educate women on cervical cancer and prevention and offer women the opportunity to self-collect HPV samples in a private designated space. CHWs would then transport HPV samples from the meeting place to a Health Centre Level 2 facility by bicycle. A driver would subsequently transport samples to a Health Centre Level 3 facility for laboratory processing. CHWs would then retrieve results from the Health Centre Level 2 facility and then deliver results to each woman’s home to offer post-test counselling to HPV-negative women and to arrange for HPV-positive women to attend a Health Centre Level 3 or above, where they would receive treatment [if eligible for cryotherapy, in accordance with WHO guidelines (World Health Organization 2013; Santesso *et al.* 2016)] or be referred for further evaluation with colposcopy and biopsy at a district hospital.

Clinic-based provider-collection was assumed to take place over two visits at a Health Centre Level 3 facility (Figure 1b). We assumed women were screened during the first visit and returned for a second visit within 2–4 weeks to obtain results; women who screened positive and were eligible received same-day cryotherapy at the results visit. Women who were ineligible for immediate cryotherapy were referred for further evaluation with colposcopy and biopsy.

Screening once in a lifetime was analysed at ages 30, 35, 40 or 45 years; to estimate the impact of screening across ages 30–49 years, we weighted the costs and health outcomes by the proportion of total screening-eligible women in Uganda in each 5-year age group (United Nations Department of Economic and Social Affairs, Population Division 2013).

Test performance, treatment and compliance parameters are displayed in Table 1. We considered population screening coverage for the self-collection campaign to be 50, 75 or 100%, and compared this to provider-collection at the same and lower coverage levels. Because compliance data are limited, we assumed comparable compliance levels for self- and provider-collection in the base case—namely, that 85% of women screened received their results, and of these, 85% of eligible women referred for treatment received cryotherapy—but varied this assumption in a sensitivity analysis.

**Table 1.** Baseline values for model variables^a^

Variable [Reference]	Baseline value
**Screening and treatment parameters**	
Screening coverage, self-collection campaign	50%, 75%, 100%
Number of women screened per monthly self-collection session (United Nations Department of Economic and Social Affairs, Population Division, 2013, World Health Organization, 2010)	
50% coverage	65
75% coverage	97
100% coverage	130
Screening coverage, clinic-based provider-collection	30% - 100%
Follow-up compliance^b^	85%
Cryotherapy compliance^b^	85%
Colposcopy compliance (among women ineligible for immediate cryotherapy)^b^	85%
Treatment compliance following colposcopy^b^	85%
careHPV test sensitivity/specificity for CIN2+	
Self-collected vaginal specimen (Jeronimo *et al*. 2014)	77%/82%
Provider-collected cervical specimen (Jeronimo *et al*. 2014)	89%/82%
Eligibility for cryotherapy (Campos *et al*. 2015a)	
No lesion or CIN1	100%
CIN2	85%
CIN3	75%
Cancer	10%
Cryotherapy effectiveness (Sauvaget *et al.* 2013, Chirenje *et al.* 2001)	92%
Colposcopy sensitivity/specificity for CIN1+^c^ (Jeronimo *et al*. 2014)	95%/51%
Cryotherapy/LEEP effectiveness following colposcopy (Chirenje *et al.* 2001)	96%
**Cost parameters, screening**^d^	
Programmatic cost per woman screened	
Self-collection campaign (50%, 75%, 100% coverage)	1.04; 0.70; 0.52
Clinic-based provider-collection	NA
Equipment cost per woman screened, self-collection campaign (50%, 75%, 100% coverage)	0.17; 0.11; 0.08
Equipment cost per woman screened, clinic-based provider-collection (Jeronimo *et al*. 2014, Mvundura and Tsu, 2014)	0.07
Direct medical/intervention cost per woman screened^e^	
Self-collection campaign (50%, 75%, 100% coverage)	9.51; 8.70; 8.28
Clinic-based provider-collection (Jeronimo *et al*. 2014, Mvundura and Tsu, 2014)	8.70
Women’s time cost per woman screened	
Self-collection campaign (50%, 75%, 100% coverage)	1.64; 1.85; 2.07
Clinic-based provider-collection (Jeronimo *et al*. 2014, Mvundura and Tsu, 2014)	3.23
**Cost parameters, diagnosis and treatment options for screen-positive women**^d^	
Direct medical costs (Mvundura and Tsu, 2014, Jeronimo *et al*. 2014)	
Cryotherapy^f^	13.49
Colposcopy^g^	7.08
Colposcopy and biopsy^g^	32.90
LEEP^f^	139.54
Direct non-medical costs^h^ (Goldie *et al*. 2005, Jeronimo *et al*. 2014)	
Self-collection campaign, immediate cryotherapy visit^i^	3.39
Clinic-based provider-collection, immediate cryotherapy visit^i^	0.34
Colposcopy/biopsy visit	17.16
LEEP/cryotherapy visit following histologic diagnosis	17.04
Treatment of local cancer (FIGO stages 1a-2a)^j^ (Goldie *et al*. 2005, Campos *et al*. 2012)	888
Treatment of regional/distant cancer (FIGO stages ≥2b)^j^ (Goldie *et al*. 2005, Campos *et al*. 2012)	1,176

a CIN, cervical intraepithelial neoplasia; FIGO, International Federation of Gynecology and Obstetrics; LEEP, loop electrosurgical excision procedure.

b Compliance is defined as the proportion of women referred to further care who comply with recommended follow-up. Follow-up compliance is the proportion of women screened who receive their results. Of those who screen positive and receive results, cryotherapy compliance is the proportion of women who receive immediate cryotherapy, if eligible. Of those who are ineligible for immediate cryotherapy, colposcopy compliance is the proportion of women who receive colposcopy. Of those with a histologic diagnosis of CIN1+, treatment compliance is the proportion who subsequently receive either cryotherapy or LEEP at a district-level facility.

c Test performance characteristics of colposcopy in START-UP were derived from the worst diagnosis of the local pathologist relative to the worst diagnosis by a quality control pathologist (gold standard); we applied the treatment threshold of CIN1+, although this was not the treatment threshold in START-UP. To derive test performance of colposcopy, we excluded histological classifications that were inadequate or with a histological classification other than negative, CIN1, CIN2, CIN3 or cancer. Because CIN1 is not a true underlying health state in the model, performance of colposcopy in the model is based on the underlying health states of no lesion, HPV infection, CIN2, or CIN3. For a treatment threshold of CIN1, we weighted sensitivity of colposcopy for women with HPV based on the country-specific prevalence of CIN1 among women with HPV infections in the START-UP studies.

d All costs are in 2011 international dollars (I$). Further details on unit cost assumptions are available in the Supplementary data S1.

e This includes the cost of the careHPV test, which was assumed to be I$5.

f Direct medical costs for follow-up procedures after treatment are presented in the Supplementary data S1.

g The proportion of colposcopies that were accompanied by a biopsy (95.6%) was drawn from START-UP data.

h Direct non-medical costs include women’s time and transportation costs. Although we assumed women walked to primary health facilities (i.e. Health Centre Level 3), and thus only incurred time costs when receiving procedures at these facilities, we assumed women used transportation to reach secondary and tertiary facilities, thus incurring transportation costs for colposcopy, treatment following a histologic diagnosis of CIN1+, or cancer treatment.

i Women’s time costs for the immediate cryotherapy visit following a self-collection campaign includes transport time, wait time, and procedure time, while provider-collection includes only procedure time, as the woman already incurred transport and wait time to receive her results. Women’s time costs for follow-up procedures after treatment are presented in the Supplementary data S1.

j All cancer costs presented include the value of women’s time spent pursuing care and transportation to health facilities.

### Cost data

We performed a micro-costing exercise in collaboration with clinical providers affiliated with the START-UP demonstration project in Uganda to estimate the resource utilization associated with a one-time self-collection campaign. Unit cost and time estimates were informed by expert opinion, budgets from a cervical cancer screening outreach programme run by the Uganda Cancer Institute, and community interventions supported by Uganda’s Infectious Disease Institute (e.g. HIV counselling and testing, male circumcision, lab sample transportation). We collected costing data in local currency units (2014 Uganda shillings) and converted these to 2011 I$ by applying GDP deflators and purchasing power parity exchange rates (World Bank, 2013). The exceptions were for the careHPV test and for equipment, which were assumed to be tradable goods; thus, we either applied official exchange rates to convert local currency units to I$ or, when costs were available in US dollars, deflated costs to 2011 levels. We annualized equipment costs with a 3% interest rate over the assumed economic lifetime of the asset, and, for equipment likely to be used for other programmes (e.g. vehicle, bicycle, megaphone), allocated only the proportion of use that would be dedicated to the campaign.

We included programmatic costs, equipment costs, direct medical costs and women’s time costs; selected costs are presented in Table 1. Programmatic costs included CHW training sessions and mobilization and outreach (i.e. public notices and radio advertisements), assuming these would take place at the district level. To allocate these costs across the number of women screened, we divided programmatic costs for the district by the estimated number of women screened (at each coverage level) in the median district size in Uganda (Geohive 2015). Equipment costs accrued for each Health Centre Level 2 catchment area included megaphones for mobilization and outreach, specimen transport boxes, a cooler with icepacks, CHW bicycles, and a car for transport of specimens from Health Centre Level 2 to the laboratory. To estimate the equipment cost per woman screened, we divided the total equipment cost by the number of women screened per catchment area over the 6-month campaign (at each coverage level). Laboratory equipment costs were drawn from the START-UP demonstration project, and were equivalent in the self-sampling campaign and clinic-based provider-collection strategies.

Direct medical and intervention costs for each monthly group self-collection session included CHW time for mobilization and outreach, conduct of the session, transport of specimens to the Health Centre Level 2, and delivery of patient results; health provider time for quality control and conduct of the session; driver time for transport of the specimens from Health Centre Level 2 to the Health Centre Level 3 laboratory; and supplies, including careHPV tests which were assumed cost I$5 per test, educational materials, gloves for CHWs to handle specimens to place in the carrier, cell phone plans and fuel. Direct intervention costs were allocated based on the number of women per monthly session, which varied by coverage level.

We included women’s time costs for time spent travelling and waiting for or receiving instruction or care, assuming women would walk to self-collection sessions (and to the Health Centre Level 3, if they screened positive).

Costs associated with clinic-based provider-collection were based on the START-UP demonstration project, and have been described elsewhere (Mvundura and Tsu 2014; Campos *et al.* 2015b). We included direct medical costs of clinical staff time, supplies, clinical equipment, laboratory staff time, laboratory supplies and laboratory equipment. Although women’s time costs were not estimated in the START-UP project, we assumed the same travel time to the Health Centre Level 3 for screening and treatment as for screen-positive women in the self-collection campaign; staff time (excluding preparation and registration time) was used as a proxy for women’s time spent on the screening procedure. Unlike the self-collection campaign strategy, programmatic cost estimates for clinic-based provider-collection were unavailable, and thus we did not include these in the base case but rather explored these in sensitivity analyses. Furthermore, costs associated with provider-collection were not assumed to vary according to population coverage level, as START-UP demonstration costs can only reflect the number of women in the study. For both the clinic-based provider-collection and self-collection strategies, estimates for time spent waiting at the clinic, as well as time costs associated with treatment and further diagnostic evaluation, were based on prior studies in Kenya and the START-UP demonstration projects (Goldie *et al.* 2005; Jeronimo *et al.* 2014).

Further details pertaining to demographics, catchment areas, and itemized costs are described in the Supplementary data S1.

### Sensitivity analyses

We explored the impact of several uncertain assumptions and inputs, including CHWs delivering screening results by text message, rather than home visits (by reducing CHW time for delivering results and women’s time for receiving results, while simultaneously increasing the proportion of the CHW monthly cell phone plan allocated for the screening programme); reducing cryotherapy compliance in the self-collection campaign strategy from 85 to 70%; decreasing or increasing prototypical district size for purposes of programmatic cost allocation (to either the 25th or 75th percentile of Ugandan district size); improving test performance of self-collection to mirror provider-collection, to reflect the similar performance of PCR-based tests regardless of collection method; and adding comparable programmatic costs to clinic-based provider-collection.

## Results

### Reduction in cancer risk

**Figure 2.**
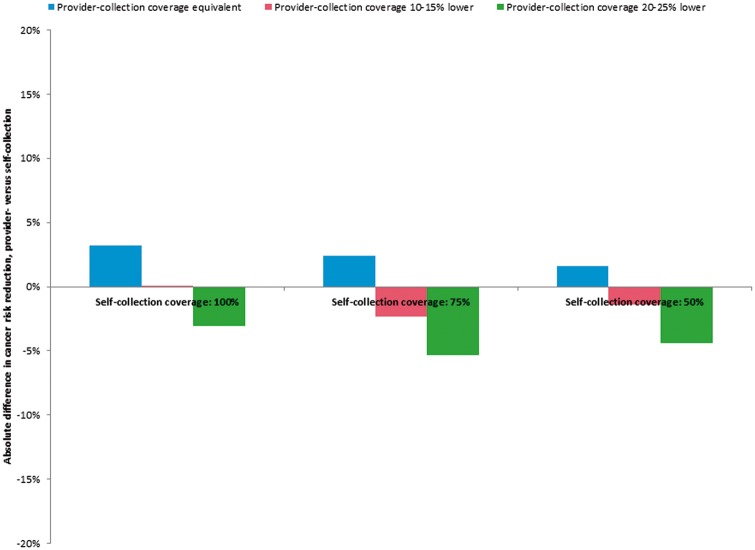
Health impact of a self-collection campaign vs clinic-based provider-collection, by population screening coverage level. The absolute difference in cancer risk reduction (clinic-based provider-collection minus self-collection campaign) is displayed on the y-axis, as population screening coverage level varies from 50 to 100% (self-collection) along the x-axis. Positive values indicate that provider-collection is the more effective strategy, while negative values indicate that the self-collection campaign is the more effective strategy. For each level of self-collection coverage, the bar on the left represents the difference in cancer risk reduction when provider-collection and self-collection coverage are equivalent; the middle bar represents the difference in risk reduction when provider-collection coverage is 10% lower (for 100 and 50% self-collection coverage; 15% lower for 75% self-collection coverage); and the bar on the right represents the difference in risk reduction when provider-collection is 20% lower (for 100 and 50% self-collection coverage; 25% lower for 75% self-collection coverage).

The relative health impact of screening once in a lifetime with either an HPV self-collection campaign or clinic-based provider-collection is presented in Table 2, and graphically in the Supplementary data S1; Figure 2 displays the absolute difference in cervical cancer risk reduction between a self-collection campaign vs provider-collection as coverage gains with self-collection increase. For each strategy, cancer risk reduction increased linearly with screening coverage. When screening coverage levels were equivalent for both strategies, clinic-based provider-collection yielded a greater mean reduction in lifetime risk of cancer than the self-collection campaign, due to enhanced sensitivity to detect precancer. At universal coverage (i.e. 100%), screening once in a lifetime between ages 30 and 49 with clinic-based provider-collection reduced cancer risk by 31.0%, whereas the self-collection campaign reduced cancer risk by 27.8%. When coverage was 75% for both strategies, clinic-based provider-collection and the self-collection campaign reduced cancer risk by 23.1 and 20.7%, respectively. As coverage declined to 50%, clinic-based provider collection reduced cancer risk by 15.4 and self-collection by 13.8%.

**Table 2.** ICERs for clinic-based provider-collection vs a self-collection campaign, by population screening coverage level^a^

**Strategy** ^b^	**Population screening coverage** ^b^	**Cancer incidence reduction, %** ^c^	**Discounted lifetime cost per woman** ^c^	**Discounted life expectancy, mean** ^c^	**ICER (I$/YLS), mean** ^c^
No screening	0%		12.44	25.20253	
Self-collection	100%	27.8	16.45	25.25676	70
Provider-collection	100%	31.0	17.36	25.26340	140
Self-collection	100%	27.8	16.45	25.25676	70
Provider-collection	90%	27.8	16.88	25.25740	670
Provider-collection	80%	24.7	16.40	25.25112	Dom
Self-collection	100%	27.8	16.45	25.25676	70
Self-collection	75%	20.7	15.59	25.24316	80
Provider-collection	75%	23.1	16.16	25.24806	120
Self-collection	75%	20.7	15.59	25.24316	80
Provider-collection	70%	21.6	15.91	25.24499	170
Provider-collection	60%	18.4	15.42	25.23878	Dom
Self-collection	75%	20.7	15.59	25.24316	80
Self-collection	50%	13.8	14.75	25.22943	Dom
Provider-collection	50%	15.4	14.92	25.23273	80
Provider-collection	40%	12.4	14.42	25.22683	80
Self-collection	50%	13.8	14.75	25.22943	130
Provider-collection	30%	9.4	13.92	25.22095	80
Self-collection	50%	13.8	14.75	25.22943	100

a Dom, dominated strategy (i.e. those that are more costly and less effective or have higher ICERs than more effective options); ICER, incremental cost-effectiveness ratio; I$, 2011 international dollars; YLS, year of life saved. Uganda per capita GDP: I$1690.

b ICERs are provided for each pair of self-collection and provider-collection population screening coverage levels; within each pair, strategies are listed in order of increasing cost; the first ICER is calculated comparing the first strategy listed within in each pair with no screening, and the second ICER is calculated relative to the first strategy listed within the pair. We assume achievable coverage with self-collection is equivalent or higher than achievable population coverage with provider-collection.

c Cancer incidence reduction for each strategy reflects percentage reduction in lifetime risk of cervical cancer compared with no screening. Cancer incidence reduction, discounted lifetime cost per woman, and discounted life expectancy represent the mean across 50 input parameter sets.

If the self-collection campaign was associated with coverage levels 10% higher than clinic-based provider-collection, reduction in cancer risk was similar between the two strategies. When coverage associated with the self-collection campaign is 100% and clinic-based provider-collection is 90%, provider-collection was similarly effective (27.8% risk reduction for both strategies). However, at a lower coverage level of 50%, self-collection yielded a more pronounced risk reduction than provider-collection at 40% coverage (13.8 vs 12.4%). When the self-collection campaign was associated with coverage gains of 15–20%, it was consistently the more effective strategy, further reducing cancer risk by an additional 2.3% [i.e. as coverage increases from 60% (provider-collection) to 75% (self-collection)] to 4.4% [i.e. coverage increases from 30 (provider-collection) to 50% (self-collection)] relative to clinic-based provider-collection.

### Cost-effectiveness analysis

The cost-effectiveness of screening once in a lifetime with an HPV self-collection campaign or clinic-based provider-collection is presented in Table 2, assuming that the self-collection campaign is associated with screening coverage equal to or greater than provider-collection coverage. When coverage was equivalent, provider-collection was associated with both higher costs (due to the intensive women’s time costs of attending the clinic twice to receive screening and results) and greater health benefits (due to enhanced detection of precancer) than the self-collection campaign. At 100% coverage, the self-collection campaign cost I$70 per year of life saved (YLS); clinic-based provider-collection was associated with a slightly higher ICER (I$140 per YLS). Both strategies would thus be considered very cost-effective, with ICERs falling well below Uganda’s per capita GDP of I$1690. As the achievable coverage with both strategies declined to 75%, the ICER associated with the self-collection campaign increased (I$80 per YLS) while clinic-based provider-collection became relatively more attractive (I$120 per YLS) as the cost per woman screened rose slightly in the self-collection campaign. At 50% coverage for both strategies, provider-collection dominated self-collection, which was no longer an efficient strategy when the costs of the campaign were spread over fewer women.

The self-collection campaign was consistently an efficient strategy when achievable coverage was greater than with clinic-based provider-collection, even when coverage gains were as small as 10%. With universal coverage of the self-collection campaign (I$70 per YLS), clinic-based provider-collection at 80% coverage was a dominated strategy. A self-collection campaign reaching 75% of the target population (I$80 per YLS) dominated clinic-based provider-collection reaching only 60% of women eligible for screening. A self-collection campaign achieving lower coverage levels of 50% (I$100 per YLS) was still more effective than clinic-based provider-collection that achieved only 30% coverage (I$80 per YLS), although it was also more costly due to the greater number of women screened and the higher cost per woman at lower campaign coverage levels.

### Sensitivity analyses

**Figure 3.**
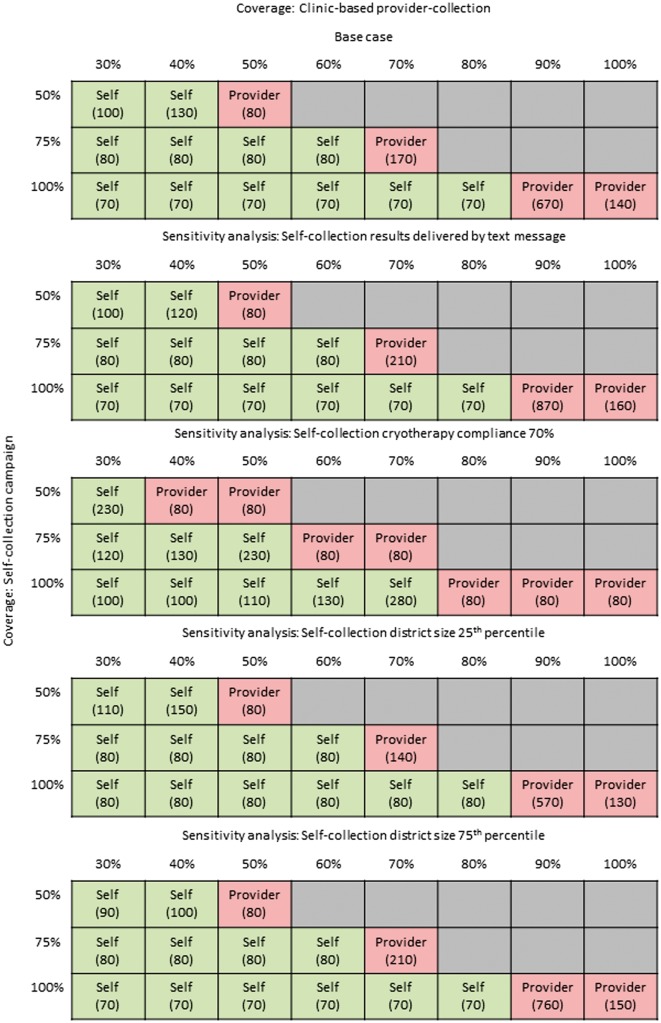

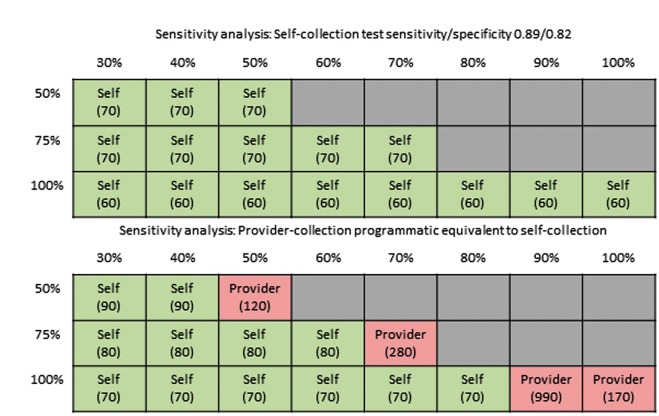
Cost-effectiveness of the self-collection campaign vs clinic-based provider-collection: base case and sensitivity analyses. The grids display the ICER for the most effective strategy with an ICER below Uganda’s per capita GDP of I$1690, as coverage associated with clinic-based provider-collection (columns) is varied between 30 and 100% and coverage associated with a self-collection campaign (rows) is varied from 50 to 100%. Empty squares indicate we did not consider a given coverage scenario, as we assumed the self-collection campaign was associated with equivalent or greater coverage than clinic-based provider-collection. The top grid displays base case results; subsequent grids represent sensitivity analysis in which the following parameters were varied independently: (1) self-collection results were delivered by text message (base case: delivered by CHW home visit); (2) self-collection cryotherapy compliance was 70% among screen-positive women who received their results (base case: 85%); (3) self-collection programmatic costs were spread across the 25th percentile district size in Uganda (base case: median district size); (4) self-collection programmatic costs were spread across the 75th percentile district size in Uganda (base case: median district size); (5) self-collection test sensitivity/specificity was equivalent to provider-collection, at 0.89/0.82 (base case: 0.77/0.82); (6) clinic-based provider-collection programmatic costs were equivalent to a self-collection campaign of comparable coverage level (base case: provider-collection had no programmatic costs). Comprehensive cost-effectiveness results are displayed in the Supplementary data S1.

Cost-effectiveness results for the base case and sensitivity analyses are presented in Figure 3, which displays the most effective strategy with an ICER below Uganda’s per capita GDP, and in the Supplementary data S1. When we assumed that CHWs delivered screening results by text message rather than home visits, the total lifetime cost per woman was reduced slightly, but ICERs for self-collection were similar to the baseline scenario; ICERs for provider-collection increased slightly as the incremental costs (compared with self-collection) rose.

When we assumed reduced compliance with cryotherapy (from 85 to 70%) in the self-collection campaign only, self-collection became a dominated strategy, unless it was associated with coverage gains of at least 20–30%.

We explored the impact of economies of scale on programmatic cost by allocating the programmatic cost per woman screened over the number of women in the 25th or 75th percentile of Ugandan districts (rather than the median district size). When there were fewer women per district (i.e. 25th percentile), the self-collection campaign was associated with slightly higher lifetime costs and ICERs, but the most effective strategy with an ICER below per capita GDP did not change relative to the base case. In larger districts, the self-collection became slightly less costly as economies of scale could be realized, but the most effective strategy with an ICER below per capita GDP did not change relative to the base case.

When we assumed test performance was the same for self- and provider-collection, the self-collection campaign dominated clinic-based provider collection and maintained an ICER of I$60 to I$70 per YLS.

Although the inclusion of comparable programmatic costs for clinic-based provider-collection increased the ICER for provider-collection, the most effective strategy with an ICER below per capita GDP did not change relative to the base case. However, unlike the base case, the self-collection campaign covering 50% of the target population dominated provider-collection with coverage <50%.

## Discussion

We estimated the health and economic impact of a one-time HPV self-collection campaign relative to clinic-based provider-collection of HPV specimens for women aged 30–49 years in Uganda. We assumed the self-collection campaign relied on CHWs to lead a series of group sessions within a primary health facility catchment area, where women would have the opportunity to self-collect HPV specimens and subsequently receive results in a home visit. To estimate the programmatic, equipment, direct medical/intervention and women’s time costs that would be incurred to implement such a campaign in Uganda, we conducted a comprehensive micro-costing exercise using data from in-country providers and existing CHW programmes. We used data from the START-UP demonstration project to inform test performance and cost data for screening and treatment procedures at the clinic. To our knowledge, this is the first study to estimate the detailed costs and long-term health benefits of HPV self-collection in a low-income country.

We found that an HPV self-collection campaign would be an effective and very cost-effective alternative to clinic-based provider-collection in Uganda, particularly at high coverage levels (i.e. 75% and above) and when self-collection is associated with greater coverage of screening-eligible women than provider-collection. Although the ICER for a self-collection campaign was relatively stable as coverage varied due to the proportionality of costs and health benefits, it is important to note that health benefits vary dramatically with coverage. A one-time self-collection campaign covering 50% of eligible women would reduce cancer risk by 14%, but this risk reduction could be doubled to 28% by increasing screening coverage to 100%. Although the attractiveness of the self-collection campaign was generally robust, if compliance with a clinic visit for cryotherapy is low among screen-positive women in a self-collection campaign, clinic-based provider-collection would be a dominant strategy unless large coverage gains could be achieved through the campaign. If HPV test performance associated with self-collection is comparable to provider-collection, the self-collection campaign would be less costly and more effective than clinic-based provider-collection.

Demonstration projects have shown that HPV self-collection can increase uptake among under-screened women in high-income countries (Castle *et al.* 2011; Arrossi *et al.* 2015; Verdoodt *et al.* 2015), and although self-collection has been found to be acceptable to women in numerous low-resource settings (Sowjanya *et al.* 2009; Levinson *et al.* 2013; Bansil *et al.* 2014; Sossauer *et al.* 2014; Rosenbaum *et al.* 2014; Mandigo *et al.* 2015; Moses *et al.* 2015), comparisons of screening uptake for self- vs provider-collection in low- and middle-income countries are limited. A randomized trial in Uganda found that 99% of women approached for self-collection of HPV specimens at home or work participated, compared with 48% of women invited to attend the clinic for screening (Moses *et al.* 2015). In our analysis, this absolute coverage gain of 50% would further decrease cancer risk by 12% [i.e. from 16% risk reduction (provider-collection, 50% coverage) to 28% risk reduction (self-collection, 100% coverage)]. A study of home-based HPV self-collection in India found that coverage relative to clinic-based screening increased from 54 to 72% (Sowjanya *et al.* 2009), while a study of home-based self-collection in Mexico found nearly perfect participation (98%), up 9% from those invited to the clinic for screening (Lazcano-Ponce *et al.* 2011). The success of door-to-door self-collection may represent the ceiling on achievable coverage, and our findings highlight the need for further evidence on the level of coverage that can be attained with the group-based campaign model for self-collection we assess here, which may facilitate a similar screening volume at lower costs. Programme architects will need to weigh the tradeoff between costs and coverage when deciding between home-based or group-based self-collection.

The cost-effectiveness of self-collection relative to provider-collection will depend not only upon achievable coverage gains, but on test performance (Arbyn and Castle 2015). We derived careHPV test performance for self-collected vaginal and provider-collected cervical specimens from the START-UP demonstration project in Uganda to reflect local conditions (Jeronimo *et al.* 2014). The relative sensitivity to detect and specificity to exclude CIN2+ were similar to values from low- and middle-income countries in a recent meta-analysis comparing self- and clinician-collected samples (Arbyn *et al.* 2014). If a low-cost PCR-based HPV test becomes available, the effectiveness and cost-effectiveness profile of self-collection will likely improve as the decrement in test sensitivity relative to provider-collection diminishes (Arbyn *et al.* 2014).

The health and economic impact of an HPV screening programme will critically depend upon linking HPV-positive women to timely and effective treatment (Gravitt *et al.* 2011). For both clinic-based provider-collection and the self-collection campaign, we assumed that 85% of women received screening results, and of these women, 85% received cryotherapy if eligible. These assumptions regarding follow-up are consistent with studies of home-based self-collection from Argentina (where women attended the health facility to receive results) (Arrossi *et al.* 2015) and slightly lower than in Peru (where women received results at home and were accompanied to the clinic by the CHW) (Levinson *et al.* 2013). However, the randomized trial in Uganda found that 53% of women who provided HPV samples could not be reached with results after three attempts by phone (Moses *et al.* 2015). Of the HPV-positive women who could be reached, 97% attended the clinic for follow-up, suggesting that in the trial setting, women were likely to seek follow-up care once they were aware of their HPV status. Our micro-costing study found that while the cost differences for CHW home visits vs text messages to deliver screening results were small and did not markedly change ICERs for self-collection, self-collection was generally a dominated strategy when compliance with cryotherapy was low. Thus, the slight increase in costs for CHW home visits to deliver results may reap substantial benefits if subsequent cryotherapy attendance is high. Whether HPV specimens are collected by a woman or her provider, programme architects will need to tailor models of care to simultaneously ensure high uptake of screening, receipt of results by all HPV-positive women, and facilitated access to timely cryotherapy among HPV-positive women in a given setting (Gravitt *et al.* 2011). Important details will include whether the most effective way to deliver results is at home, via phone call, or via text message. Operational factors such as minimizing time to results delivery or having a woman programme the CHW’s phone number into her phone (so she will later recognize that she is being contacted regarding results) may be critical (Moses *et al.* 2015). Although linkage of screen-positive women to timely cryotherapy is essential to an effective programme, it will likely impose burdens on the health care system as access to screening improves. New treatment technologies currently being tested in low-resource settings—including ablative technologies that are portable and do not require gas—may play an important role in lessening the burden of treating all HPV-positive women if feasibility and cost-effectiveness can be demonstrated.

There are several limitations to this analysis. Because our model for a self-collection campaign is hypothetical, it may not represent a feasible and acceptable approach in Uganda. Although we attempted to mitigate this limitation by working with in-country providers, further collaboration with stakeholders in Uganda would be needed to refine and test the model of care we propose. Furthermore, our micro-costing exercise may not have identified all necessary components of the programme. Although we collaborated with in-country providers to estimate the proportion of equipment allocated for a self-collection campaign, the duration of requisite CHW training, the amount of CHW and woman time that would be required during a group self-collection session, and other measures, these may over- or underestimate real-world costs of such a campaign. We assumed laboratory processing of careHPV tests could occur at Health Centre Level 3 facilities, when in actuality greater constraints on the number of careHPV machines might lead to increased costs for laboratory transport to other facilities for both self- and provider-collection strategies. Our assumptions regarding the number of screening-eligible women in a prototypical health facility catchment area and district represent average or median sizes for Uganda, and thus costs for self-collection may be different in more or less densely populated areas. However, in the absence of data from resource-intensive implementation, geospatial, and time and motion studies, we believe our costing assumptions are reasonable estimates for a country-wide campaign.

Our costing estimates for clinic-based provider-collection and for treatment were drawn from the START-UP demonstration project in Uganda, and thus represent real-world data, albeit in the context of a study setting. We did not include programmatic costs for scale-up of provider-collection, even though this would also be a new intervention if scaled throughout Uganda. Due to limited data, we also did not account for changes in costs based on volume at the clinic that would accompany changes in coverage of the target population. However, we did perform a sensitivity analysis in which we included programmatic costs for provider-collection, and results were generally consistent with our base case analysis except at lower levels of coverage (i.e. 50%), when economies of scale were not achieved.

In addition to limitations surrounding our costing assumptions, we did not consider the possibility of differential risk of HPV infection and cervical cancer in women with access to clinic-based provider-collection vs those targeted by a self-collection campaign. A recent study from the Netherlands found that non-attendees of the screening programme who responded to self-collection had a higher risk of CIN2 and CIN3 relative to women who participated in regular screening (Gok *et al.* 2012). If uptake of self-collection in Uganda is higher among women without access to clinic-based screening who thus may be at an elevated risk of cancer, the cost-effectiveness profile will likely become more attractive due to averted cancer costs and cases. We also did not consider the impact of screening in women who have been previously vaccinated against HPV. Given that the HPV vaccination programme in Uganda is in its nascent stages, and that there are two to three generations of women at risk for cervical cancer who are past the target age for adolescent vaccination, our objective was to evaluate the impact of HPV-based screening in an unvaccinated population. As vaccinated cohorts age and more data on the duration of HPV vaccine protection become available, it will be important to re-evaluate screening protocols (including optimal start age, frequency and interval) in vaccinated cohorts and in the general population, depending on vaccination coverage (Campos *et al.* 2012).

As screening programmes are implemented and scaled in low- and middle-income settings, countries will need to design programmes based on many factors in addition to the cost-effectiveness profile of a screening strategy, including acceptability, feasibility, existing infrastructure, anticipated gains relative to competing healthcare priorities and affordability. The chronic shortage of healthcare workers in low-resource settings will likely be a barrier to scale-up of clinic-based screening by a trained provider. By shifting the task of screening to CHWs and women, a model of care involving self-collection of HPV specimens has the potential to circumvent provider shortages and concentrate limited provider time on treatment of screen-positive women. Given the importance of screening coverage and the potential for increased uptake with self-collection, programmes may achieve greater health benefits by tailoring programmes to different segments of the population than by offering a one-size-fits-all approach. In Uganda, for instance, where 84% of the population lives in rural areas (World Bank 2013), a widespread self-collection campaign may achieve economies of scale and reach the vast majority of screening-eligible women. More densely populated urban areas might efficiently rely predominantly on clinic-based provider-collection, with campaigns selectively targeted toward women without access to routine health care. The comparative- and cost-effectiveness results presented here provide reassurance that both strategies are likely associated with similar costs and health benefits in the Ugandan setting, so long as high coverage and linkage to treatment are achieved. However, it is important to note that information on the value for money is not equivalent to affordability or the financial impact of a programme on a payer’s budget (Marseille *et al.* 2015). Both the cost-effectiveness profile and recurrent financial costs must be favourable to implement a sustainable screening programme. Decision makers will need to examine the programmatic investments that will be necessary to scale up infrastructure and train personnel for screening and treatment, and how these investments may compete or yield synergies with other health interventions under consideration.

Following a detailed analysis of resource utilization for a hypothetical one-time HPV self-collection campaign in Uganda, we found that a campaign relying on CHWs would be very cost-effective, reducing cancer risk by >20% if coverage reaches 75%. We are not aware of any other studies reporting the costs and long-term benefits of an HPV self-collection strategy in a low-income country, and additional data from demonstration projects will be needed to confirm the validity of our logistical, costing and compliance assumptions, as well as the feasibility and acceptability of such a model of care in Uganda or other low-income settings. From a health systems perspective, a self-collection campaign may provide an opportunity to strengthen the workforce of CHWs, offering valuable training and experience relevant to the provision of other preventive interventions in settings with few health care providers, while enabling a high volume of women to receive screening to prevent the leading cause of cancer death in Eastern Africa (Ferlay *et al.* 2013). We hope this analysis will inform funding decisions and programmatic planning to implement and scale HPV-based cervical cancer prevention in low- and middle-income settings where the burden of cervical cancer is highest.

## Supplementary data

Supplementary data are available at *HEAPOL* online.

## Supplementary Material

Supplementary Appendix
