# Supplementary material for: Cost-effectiveness of an HPV self-collection campaign in Uganda: comparing models for delivery of cervical cancer screening in a low-income setting
Source: Health Policy Plan. 2017 Feb 20;32(7):956–68. doi: 10.1093/heapol/czw182 (PMC5886074; doi:10.1093/heapol/czw182)
Supplement: Supplementary Appendix [file appendix_costing_assumptions_czw182.docx]

**Cost-Effectiveness of an HPV Self-Collection Campaign in Uganda: Exploring Models for Delivery of Cervical Cancer Screening in a Low-Income Setting**

**SUPPLEMENTARY APPENDIX**

**Supplementary Methods: Model Calibration Results**

Model fit to empirical data on age-specific high-risk HPV prevalence data from the START-UP project and age-specific cancer incidence in Uganda is displayed in **Appendix Figures 1 and 2**.

**Supplementary Methods: Cost Data**

**Screening Process**

HPV self-collection of vaginal samples in Uganda is limited to pilot and demonstration projects [[1](#_ENREF_1),[2](#_ENREF_2)], and costing data are limited for models of care that involve self-collection in a community setting. Our objective was to conduct a micro-costing study of community-based HPV self-collection in Uganda in order to inform costing inputs for an individual-based microsimulation model of HPV infection and cervical cancer, which we then used to evaluate the cost-effectiveness of a group-based HPV self-collection campaign.

Working with clinical experts and providers with experience in Uganda, we designed one potential model of screening and treatment and micro-costed each required component for the process for a prototypical village in Uganda. We assumed a onetime campaign for women aged 30 to 49 years would consist of several monthly group sessions in a village, facilitated by community health workers (CHWs). Following mobilization efforts, a group session would be held in a local setting, during which a health provider and CHWs would educate women on cervical cancer and prevention and offer women the opportunity to self-collect HPV samples. CHWs would then transport HPV samples from the meeting place to a Health Center Level 2 facility by bicycle. A driver would subsequently transport samples to a Health Center Level 3 facility for laboratory processing. CHWs would then deliver results to each woman’s home to offer post-test counseling to HPV-negative women and to arrange for HPV-positive women to attend a Health Center Level 3 or above for treatment or further evaluation.

**Structure of the Self-Sampling Campaign**

Our individual-based microsimulation model projects lifetime costs and health outcomes associated with cervical cancer prevention strategies for a hypothetical cohort of women. Our current modeling tools are calibrated to epidemiologic data from Uganda, and are not intended to incorporate demographic data or to project the country-wide budgetary impact of an intervention. The model uses data on the per-woman costs of a group session and subsequent clinical encounters. Thus, all costs for group sessions— including mobilization, delivery of results, programmatic, and equipment costs— need to be allocated based on the number of women screened.

To establish the number of screening-eligible women in a prototypical catchment area for allocating the per-woman costs of each monthly session, as well as to determine a reasonable size and number of monthly sessions to comprise a campaign with broad coverage, we determined the number of women of screening age (30 to 49 years) in Uganda in 2015 [[3](#_ENREF_3)]. We divided this total screening eligible population by the total number of Tier 1 facilities (defined by the World Health Organization as health centers or health posts: n=4,337) in Uganda [[4](#_ENREF_4)] to derive the number of women (n=780) in a catchment area for each Tier 1 facility. To ensure feasible size and quality control of monthly sessions, we assumed a campaign would need to include 6 monthly sessions, ranging in size from 65 to 130 women, depending on the success of mobilization efforts.

To establish the number of screening-eligible women in a prototypical district for allocating programmatic costs that would be incurred at the district level, we used preliminary census data from 2014 to determine the median district size in Uganda (n=243,876)[[5](#_ENREF_5)]. Assuming the proportion of screening-eligible women in the total population is uniform for each district at around 8.7%, we estimated the number of screening-eligible women in a prototypical district (n=21,134). Given the ratio between the number of screening-eligible women area in a prototypical district to the number of screening-eligible women in a prototypical Tier 1 facility catchment area, we estimated there would be approximately 27 Tier 1 facilities within a prototypical district.

Assumptions and figures pertaining to the structure and size of the self-sampling campaign are shown in **Appendix** **Table 1.**

**Micro-Costing Study of Group-Based Self-Sampling in the Community**

We consulted with clinical experts and providers with experience in Uganda to estimate the costs of a potential campaign with 6 consecutive monthly group sessions for self-collection in each prototypical catchment area. Unit cost and time estimates were informed by expert opinion, budgets from a cervical cancer screening outreach program run by the Uganda Cancer Institute, and community interventions supported by the Infectious Disease Institute (Uganda) such as HIV counseling and testing, male circumcision, and lab sample transportation.

We collected costing data in local currency units (2014 UGX) and converted these to 2011 international dollars (I$) by 1) applying GDP deflators to deflate to year 2011 levels; and 2) applying purchasing power parity (PPP) exchange rates [[6](#_ENREF_6)]. The exceptions were for the careHPV test and for equipment, which were assumed to be tradable goods. For these tradable goods, one international dollar is equivalent to one US$, so we either used official exchange rates to convert local currency units (2011) to international dollars (2011) or, when costs were available in US$, deflated US$ to 2011 levels [[6](#_ENREF_6)].

*Programmatic costs of campaign*

Programmatic costs included 1) onetime CHW training sessions; and 2) public notices and radio advertisements for outreach and mobilization. We assumed these costs would be incurred at the district level, and thus per-woman programmatic costs were derived based on the number of women screened in the prototypical district throughout the 6 month campaign, assuming population coverage was either 50%, 75%, or 100%. Resource requirements for a CHW training session included CHW time, trainer time, printed materials, and refreshments. Public notices and radio advertisements within the district were assumed to occur for the duration of the campaign (i.e., 6 months). Programmatic costs are shown in **Appendix** **Table 2.**

*Direct medical/intervention costs per monthly group self-sampling session*

We estimated the costs of each monthly session in a prototypical catchment area, including 1) CHW time (for mobilization and outreach, conduct of the session, transportation of specimens to the Health Center Level 2, and delivering patient results); 2) health provider time (for quality control and conduct of the session); 3) driver time (for transport of the specimens from Health Center Level 2 to Health Center Level 3 for processing); and 4) supplies (careHPV test kits, educational materials, gloves for CHWs, cell phone plans, fuel for specimen transport). Direct medical/intervention costs were allocated based on the number of women per monthly session, and are shown in **Appendix** **Table 3.**

*Equipment costs*

For monthly group self-collection sessions within a catchment area, we included equipment costs for 1) 2 megaphones used for mobilization and outreach; 2) 2 specimen transport boxes; 3) a cooler with icepacks; 4) 2 CHW bicycles; and 5) a car for transport of specimens from Health Center Level 2 to the laboratory. We assumed all equipment items were tradable goods, and accordingly converted local currency units to international dollars using the official exchange rate [[6](#_ENREF_6)]. We annualized equipment costs with a 3% interest rate, and assumed an economic life of 3 years for the bicycles, transport boxes, and cooler/icepacks and 5 years for the megaphones and car. We assumed annual maintenance costs included new batteries for the megaphone and quarterly maintenance for the bicycles and car. Given the 6 month duration of the self-sampling campaign, we assumed 25% (in the case of the megaphones, transport boxes, cooler, and bicycles) and 0.1% of annualized costs (in the case of the car) would be allocated to the campaign. We calculated an equipment cost per woman screened based on the proportion of the annualized cost of the equipment that was used for the self-sampling campaign and the number of women screened per catchment area over the 6 month campaign, under different coverage scenarios (**Appendix** **Table 4**).

*Women’s time costs*

We derived women’s time costs from the United Nations Development Programme Human Development Indicator, “Estimated GNI per capita, female”, which was derived from the ratio of female to male wage, female and male shares of economically active population, and gross national income (GNI) and reported in constant 2011 I$ [[7](#_ENREF_7)]. We assumed this represented annual income for working 40 hours per week, 50 weeks per year to estimate an average hourly wage. We included women’s time costs for traveling, as well as for waiting for or receiving instruction or care. We assumed women would walk to self-sampling sessions and the clinic (to receive care if they screened positive). Women’s time and transportation costs pertaining to group-based self-sampling are shown in **Appendix** **Table 5**.

For care at a health facility (for screen-positive women), estimates for time spent traveling, waiting, and receiving care was dependent upon the facility level where care was assumed to take place (**Appendix** **Table 6**). Women’s time estimates for waiting were obtained from prior studies in Kenya (**Appendix** **Table 7**) [[8](#_ENREF_8),[9](#_ENREF_9)]. Estimates of women’s time spent receiving a procedure were based on site-specific data from the Screening Technologies to Advance Rapid Testing for Cervical Cancer Prevention–Utility and Program Planning (START-UP) demonstration projects, with staff time spent on the procedure (excluding preparation and registration time, which we assumed were built into patient waiting time) used as a proxy for women’s procedure time.

**Comparator: Provider-Sampling at the Clinic**

We have previously evaluated the cost-effectiveness of careHPV (provider-collection) taking place at the clinic [[10](#_ENREF_10)]. This analysis used the direct medical costs of screening, diagnosis, and treatment of precancerous lesions from the START-UP demonstration study in Kampala, Uganda. Direct medical costs included clinical staff time, clinical supplies, drugs, clinical equipment, laboratory staff time, laboratory supplies, and laboratory equipment and are shown in **Appendix** **Table 8**. As with our costing methodology for self-sampling in group sessions (described above), all costs are in 2011 I$ and equipment was assumed to be tradable.

To be consistent with women’s time estimates gathered from in-country experts for self-sampling, we assumed comparable estimates of women’s travel time to the clinic for screening and/or treatment.

**Protocols for Treatment of Precancer**

Women who screened positive who were ineligible for cryotherapy were assumed to be referred to a secondary facility for colposcopy and subsequent treatment. Treatment protocols were based on information from in-country clinicians familiar with standard of care and availability of and preferences for treatment options. We assumed that, upon a histologic diagnosis of CIN1, women received cryotherapy at a secondary facility; a histologic diagnosis of CIN2/3 was followed by cryotherapy for approximately 80% of women, and LEEP for approximately 20% of women, and treatment occurred at a secondary facility.

Following treatment of precancerous lesions with either cryotherapy or LEEP, we assumed the setting-specific follow-up protocols as used in the START-UP demonstration studies (**Appendix** **Table 9**). We included direct medical costs of each procedure (**Appendix** **Table 8**), as well as women’s time and transportation costs. While women in the START-UP studies could be seen prior to scheduled follow-up visits as necessary, we did not have data on these unscheduled visits. Treatment complications in each site were very rare, so we did not consider these costs in the base case analysis.

**Cost of Cancer Care by Stage**

Costs associated with cancer care by stage (Local vs. Regional or Distant), including direct medical costs, women's time costs for time spent receiving care, women's transportation costs to health facilities, and cancer staging costs were derived from previous analyses and converted to 2011 I$ as described above. Cancer care costs in Uganda were based on primary data from Kenya, as we have previously described [[9](#_ENREF_9),[11](#_ENREF_11)] (**Appendix** **Table 8)**.

**Supplementary Results**

Incremental cost-effectiveness ratios for sensitivity analyses— exploring the impact of delivering screening results by test message; reducing cryotherapy compliance in the self-collection campaign strategy; decreasing or increasing prototypical district size for purposes of programmatic cost allocation; improving test performance of self-collection; and adding comparable programmatic costs to clinic-based provider-collection— at different levels of relative screening coverage are displayed in **Appendix** **Tables 10-15.**

**Appendix** **Table 1. Uganda: population and capacity estimates for group-based monthly HPV self-collection sessions. References in parenthesis.**

| **Variable** | | **Number** |
| --- | --- | --- |
| Total population, Uganda [[3](#_ENREF_3)] | | 39,032,383 |
| Screening-eligible women, Uganda^a^ [[3](#_ENREF_3)] | | 3,382,547 |
| Screening-eligible women, district level^b^ | |  |
| 25th percentile | | 15,470 |
| Median | | 21,134 |
| 75th percentile | | 33,670 |
| Total Tier 1 health facilities^c^ [[4](#_ENREF_4)] | | 4,337 |
| Screening-eligible women per Tier 1 health facility^d^ | | 780 |
| Women screened per monthly session^e^ | |  |
| 100% coverage | | 130 |
| 75% coverage | | 97 |
| 50% coverage | | 65 |
| Tier 1 health facilities per district^f^ | |  |
| 25th percentile | | 20 |
| Median | | 27 |
| 75th percentile | | 43 |
| ^a^ | Total women aged 30 to 49 years in Uganda, 2015. | |

^b^  Assumes proportion of screening-eligible women in the total population (~8.7%) is uniform across all districts. Percentile refers to district size. Figures are derived from age-structured and district-level population data [[3](#_ENREF_3),[5](#_ENREF_5)].

^c^ Tier 1 health facilities, as defined by the World Health Organization, include health centers and health posts.

^d^  Women aged 30 to 49 years per Tier 1 health facility is the total number of screening eligible women in Uganda divided by the total number of Tier 1 health facilities. We refer to this as the number of women in a prototypical catchment area.

^e^ Assumes 6 consecutive monthly sessions to cover the catchment area during the campaign, with the number of women screened in each session varying according to coverage level.

^f^ The ratio of screening-eligible women in district to screening-eligible women per Tier 1 health facility is a proxy for the number of Tier 1 facilities in a prototypical district. Percentile refers to district size.

**Appendix** **Table 2. Programmatic figures and costs for a group-based monthly self-collection campaign.**^a^

| **Variable** | | **Number** |
| --- | --- | --- |
| **CHW training sessions (district-level)** | | |
| Number of CHWs per district^b^ | | 54 |
| Hours per session | | 5 |
| Sessions per district | | 1 |
| CHW hourly wage | | I$1.45 |
| Printed materials^c^ | | I$1,008 |
| Refreshments^c^ | | I$151 |
| CHW time^c^ | | I$394 |
| Trainer fee/stipend | | I$85.56 |
|  | |  |
| **Public notices/radio advertisements** | | |
| Cost of notices/advertisements for 6 months | | I$9,329 |
|  | |  |
| **Total programmatic costs per woman screened** | | |
| Population screening coverage^d^  50%  75%  100% | | I$1.04  I$0.70  I$0.52 |
| ^a^ | CHW: community health worker; I$: 2011 international dollars. | |

^b^ Assumes 2 CHWs per Tier 1 health facility catchment area.

^c^  Based on number of community health workers per session.

^d^ Based on number of women screened at the district level, assuming screening coverage varies from 50% to 100%.

**Appendix** **Table 3. Direct medical costs for group-based monthly self-collection sessions.^a^**

| **Variable** | | **Number** |
| --- | --- | --- |
| **CHW time per session** | | |
| Number of CHWs per catchment area | | 2 |
| CHW hourly wage | | I$1.45 |
| Time, mobilization and outreach | | 5 hours |
| Time, monthly session^b^ | | 2.08 – 3.17 hours |
| Time, transporting specimens to Health Center Level 2 | | 45 minutes |
| Time, obtaining results from Health Center Level 2 | | 15 minutes |
| Time, traveling to deliver results (per woman) | | 20 minutes |
| Time, delivering results (per woman)^c^ | | 11 minutes |
|  | |  |
| **Other personnel** | | |
| Provider fee/stipend (for quality control of session) | | I$85.56 |
| Driver, for transport of specimens from Health Center 2 to laboratory | | I$23.25 |
| Laboratory staff^d^ | | I$0.16 per sample |
|  | |  |
| **Supplies** | | |
| careHPV test kit (per woman) | | I$5 |
| Handout (per woman) | | I$0.46 |
| Laminated posters for collection rooms^e^ | | I$1.39 |
| Gloves for 2 CHWs handling tubes | | I$0.93 |
| Fuel for specimen transport to laboratory^f^ | | I$4.42 |
| Monthly cell phone plan, CHW^g^ | | I$31.11 |
| Monthly cell phone plan, driver^g^ | | I$1.56 |
| Laboratory supplies^d^ | | I$0.63 per sample |
| Laboratory equipment^d, h^ | | I$0.26 per sample |
|  | |  |
| **Total direct medical costs per woman screened** | | |
| Population screening coverage^i^  50%  75%  100% | | I$9.51  I$8.70  I$8.28 |
| ^a^ | CHW: community health worker; I$: 2011 international dollars. | |

^b^  Based on number of women per session (varies from 65 to 130, as screening coverage varies from 50% to 100%).

^c^ Cost of post-test counseling only accrued if woman was at home.

^d^ From the START-UP demonstration project.

^e^ Assumes 3 posters, which are re-used for each monthly session over the course of the campaign.

^f^ Assumes 30 minutes of driving time.

^g^ Assumes 50% of CHW cell phone use and 2.5% of driver cell phone use is dedicated to the self-sampling campaign per month, assuming a 40 hour work week.

^h^ Laboratory equipment costs include a storage freezer, Uninterrupted Power Supply plus, computer, and timer.

^i^ Based on number of women per monthly session, as screening coverage varies from 50% to 100%.

**Appendix** **Table 4. Equipment costs and assumptions for a group-based monthly self-collection campaign.^a^**

| **Variable** | | **Number** |
| --- | --- | --- |
| Megaphone | |  |
| Cost | | I$41.14 |
| Number required | | 2 |
| Economic life, years | | 5 |
| Annual maintenance (8 C batteries) | | I$13.80 |
| Proportion of year spent on 6 month self-sampling campaign | | 0.25 |
| Annualized cost allocated to self-sampling | | I$11.39 |
|  | |  |
| Bicycle with box | |  |
| Cost | | I$119 |
| Number required | | 2 |
| Economic life, years | | 3 |
| Annual maintenance | | I$1.27 |
| Proportion of year spent on 6 month self-sampling campaign | | 0.25 |
| Annualized cost allocated to self-sampling | | I$17.47 |
|  | |  |
| Car | |  |
| Cost | | I$55,307 |
| Number required | | 1 |
| Economic life, years | | 5 |
| Annual maintenance | | I$737 |
| Proportion of year spent on 6 month self-sampling campaign | | 0.001 |
| Annualized cost allocated to self-sampling | | I$18.48 |
|  | |  |
| Specimen transport box | |  |
| Cost | | I$93.51 |
| Number required | | 2 |
| Economic life, years | | 3 |
| Annual maintenance | | 0 |
| Proportion of year spent on 6 month self-sampling campaign | | 0.25 |
| Annualized cost allocated to self-sampling | | I$12.14 |
|  | |  |
| Cooler and icepacks | |  |
| Cost | | I$24.69 |
| Number required | | 1 |
| Economic life, years | | 3 |
| Annual maintenance | | 0 |
| Proportion of year spent on 6 month self-sampling campaign | | 0.25 |
| Annualized cost allocated to self-sampling | | I$2.18 |
|  | |  |
| **Total equipment costs per woman screened** | | |
| Population screening coverage^b^  50%  75%  100% | | I$0.17  I$0.11  I$0.08 |
| ^a^ | CHW: community health worker; I$: 2011 international dollars. Annualized costs converted from local currency to I$ using official exchange rates. Discount rate = 3%. | |

^b^  Based on number of women screened per catchment area throughout the 6 month campaign (as screening coverage varies from 50% to 100%).

**Appendix** **Table 5. Women’s time costs and figures for group-based monthly self-collection sessions and follow-up.**^a^

| **Variable** | | **Number** |
| --- | --- | --- |
| Hourly wage | | I$0.68 |
|  | |  |
| **Women’s time, self-collection process** | | |
| Time, round-trip travel to group session | | 40 minutes |
| Time, instruction | | 1 hour |
| Time, waiting to self-collect^b^ | | 38 – 76 minutes |
| Time, self-collection and delivery of specimen to CHW | | 7 minutes |
| Time, receiving results and post-test counseling | | 11 minutes |
|  | |  |
| **Women’s time, if screen-positive** | | |
| Time, round-trip travel to Health Center Level 3 for cryotherapy | | 3 hours |
| Time, waiting for cryotherapy | | 1.5 hours |
| Time, receiving cryotherapy | | 30 minutes |
|  | |  |
|  | |  |
| **Total cost per woman screened** | | |
| Population screening coverage^c^  50%  75%  100% | | I$1.64  I$1.85  I$2.07 |
| ^a^ | CHW: community health worker; I$: 2011 international dollars. | |

^b^  Wait time varies based on the number of women in attendance (varies from 65 to 130 women).

^c^  Based on number of women screened per session, assuming screening coverage varies from 50% to 100%. Total only includes the costs associated with initial screening, and does not include the value of time receiving results and post-test counseling, which depends upon availability during CHW visits.

**Appendix** **Table 6. Location of Service Delivery for Screening, Diagnosis, and Treatment of Precancerous Lesions and Cancer.**^a^

| **Procedure** | | **Location of services** |
| --- | --- | --- |
| HPV DNA test (self-sampling) | | Group session at community meeting place |
| HPV DNA test (provider-sampling) | | Primary Health Center Level 3 |
| Cryotherapy | | Primary Health Center Level 3 (for women eligible for screen-and-treat cryotherapy)  Secondary facility (for women ineligible for screen-and-treat cryotherapy |
| Colposcopy/biopsy | | Secondary facility |
| LEEP | | Secondary facility |
| Follow-up visits (after cryotherapy or LEEP) | | Primary Health Center Level 3 (for examinations and Pap)  Secondary facility (if colposcopy is necessary) |
| Cancer treatment | | Tertiary facility |
| ^a^ | HPV: human papillomavirus; LEEP: loop electrosurgical excision procedure; VIA: visual inspection with acetic acid. | |

**Appendix** **Table 7. Women’s Time Spent Receiving Care at a Health Care Facility.**^a^

| **Procedure** | | **Time Spent Receiving Care (Minutes)** | | | |
| --- | --- | --- | --- | --- | --- |
|  | |  |  |  | **Uganda[**[**2**](#_ENREF_2)**,**[**9**](#_ENREF_9)**]** |
| **Clinic-based screening** | | | | | |
| Wait time  Procedure time  Transport time (round-trip) | | |  |  | 90 |
|  |  |  |  |  | 15 |
|  |  |  |  |  | 180 |
| Receiving results (negative)^b^  10 | | | | | |
| Receiving results (positive) 15 | | | | | |
| **Diagnosis** | | | | | |
| Wait time | | |  |  | 180 |
| Procedure time  Transport time (round-trip) | | |  |  | 35 |
|  |  |  |  |  | 340 |
|  | | | | | |
| **Treatment of Precancer: Screen-and-Treat Cryotherapy** ^c^ | | | | | |
| Wait time  Procedure time  Transport time (round-trip) | | |  |  | 180 |
|  |  |  |  |  | 30 |
|  |  |  |  |  | 180 |
|  | | | | | |
| **Treatment of Precancer: LEEP** | | | | | |
| Wait time  Procedure time  Transport time (round-trip) | | |  |  | 180 |
|  |  |  |  |  | 25 |
|  |  |  |  |  | 340 |
| ^a^ | I$: international dollars. LEEP: loop electrosurgical excision procedure. | | | | |
| ^b^ | Applicable to careHPV and Pap screening. | | | | |
| ^c^ | We assumed eligible women received cryotherapy in the same visit they received screening results, if screened at the clinic. For women who received cryotherapy following diagnostic confirmation of CIN, wait time and transport time were the same as for LEEP, as cryotherapy was assumed to take place at a secondary facility. | | | | |

**Appendix** **Table 8. Direct Medical and Non-Medical Costs, Screening and Treatment of Precancer and Cancer.**^a^

| **Variable** | | **Cost (2011 I$)** |
| --- | --- | --- |
| **Direct medical costs, screening and treatment of precancer[**[**2**](#_ENREF_2)**,**[**12**](#_ENREF_12)**]** | | |
| careHPV (cervical specimen) | | 8.78 |
| Cytology | | 12.25 |
| Visual exam | | 2.90 |
| Colposcopy | | 7.08 |
| Colposcopy and biopsy | | 32.90 |
| Cryotherapy | | 13.49 |
| LEEP | | 140 |
|  | |  |
| **Direct non-medical costs [**[**9**](#_ENREF_9)**,**[**11**](#_ENREF_11)**]** | | |
| Transportation (round-trip, secondary facility) | | 10.87 |
|  | |  |
| **Treatment of cancer (direct medical and non-medical costs)[**[**9**](#_ENREF_9)**,**[**11**](#_ENREF_11)**]** | | |
| Local cancer (FIGO stages 1a-2a) | | 888 |
| Regional/distant cancer (FIGO stages ≥2b | | 1176 |
| ^a^ | I$: 2011 international dollars. Direct medical costs include equipment costs. | |

**Appendix** **Table 9. Follow-up Protocols after Treatment of Precancerous Lesions.**^a^

| **Treatment** | |  |  |  | **Follow-up Protocol** |
| --- | --- | --- | --- | --- | --- |
| **Cryotherapy** | | | | | |
|  | |  |  |  | 6 week exam |
|  | |  |  |  | 1 year Cytology/Colposcopy^b^ |
|  | | | | | |
| **LEEP** | | | | | |
|  | |  |  |  | 6 week exam |
|  | |  |  |  | 1 year Cytology  1 year Colposcopy, as needed^b^ |
| a | LEEP: loop electrosurgical excision procedure. Follow-up protocols were based on the START-UP demonstration study. We included direct medical costs and women’s time and transportation costs for each procedure. A 6 week visual exam was associated with the same costs as VIA at the primary facility. Cytology was assumed to take place at a primary facility, while colposcopy was assumed to take place at a secondary facility. | | | | |
| b | Colposcopy at 1 year was performed as needed in Uganda. Approximately 15% of women who received treatment required a colposcopy and biopsy at 1 year for suspected recurrence. | | | | |

**Appendix** **Table 10. Sensitivity analysis: Screening results delivered by text message (base case: home visit by CHW). Incremental cost-effectiveness ratios for clinic-based provider-collection versus a self-collection campaign, by population screening coverage level.^a^**

| **Strategy**^b^ | **Population screening coverage**^b^ | **Discounted lifetime cost per woman**^c^ | **Discounted life expectancy, mean**^c^ | **ICER (I$/YLS), mean**^c^ |
| --- | --- | --- | --- | --- |
| Natural history | 0% | 12.44 | 25.20253 | -- |
|  |  |  |  |  |
| Self-collection | 100% | 16.32 | 25.25676 | 70 |
| Provider-collection | 100% | 17.36 | 25.26340 | 160 |
|  |  |  |  |  |
| Self-collection | 100% | 16.32 | 25.25676 | 70 |
| Provider-collection | 90% | 16.88 | 25.25740 | 870 |
|  |  |  |  |  |
| Self-collection | 100% | 16.32 | 25.25676 | 70 |
| Provider-collection | 80% | 16.40 | 25.25112 | Dom |
|  |  |  |  |  |
| Self-collection | 75% | 15.52 | 25.24316 | 80 |
| Provider-collection | 75% | 16.16 | 25.24806 | 130 |
|  |  |  |  |  |
| Self-collection | 75% | 15.52 | 25.24316 | 80 |
| Provider-collection | 70% | 15.91 | 25.24499 | 210 |
|  |  |  |  |  |
| Provider-collection | 60% | 15.42 | 25.23877 | Dom |
| Self-collection | 75% | 15.52 | 25.24316 | 80 |
|  |  |  |  |  |
| Self-collection | 50% | 14.73 | 25.22943 | Dom |
| Provider-collection | 50% | 14.92 | 25.23273 | 80 |
|  |  |  |  |  |
| Provider-collection | 40% | 14.42 | 25.22683 | 80 |
| Self-collection | 50% | 14.73 | 25.22943 | 120 |
|  |  |  |  |  |
| Provider-collection | 30% | 13.92 | 25.22095 | 80 |
| Self-collection | 50% | 14.73 | 25.22943 | 100 |

^a^ CHW: community health worker; Dom: dominated strategy ( i.e., those that are more costly and less effective or have higher ICERs than more effective options); ICER: incremental cost-effectiveness ratio; I$: 2011 international dollars; YLS: year of life saved. Uganda per capita GDP: I$1,690. When CHWs delivered results by text message instead of in person, costs associated with CHW time went down, but the proportion of monthly cell plan usage increased from 50% (base case) to 100%.

^b^ ICERs are provided for each pair of self-collection and provider-collection population screening coverage levels; within each pair, strategies are listed in order of increasing cost. We assume achievable coverage with self-collection is equivalent or higher than achievable population coverage with provider-collection. Natural history refers to a scenario with no screening.

^c^ Discounted lifetime cost per woman and discounted life expectancy represent the mean across 50 input parameter sets.

**Appendix** **Table 11. Sensitivity analysis: Cryotherapy compliance 70% for self-collection campaign, 85% for provider-collection (base case: 85% for both self- and provider-collection). Incremental cost-effectiveness ratios for clinic-based provider-collection versus a self-collection campaign, by population screening coverage level.^a^**

| **Strategy**^b^ | **Population screening coverage**^b^ | **Discounted lifetime cost per woman**^c^ | **Discounted life expectancy, mean**^c^ | **ICER (I$/YLS), mean**^c^ |
| --- | --- | --- | --- | --- |
| Natural history | 0% | 12.44 | 25.20253 | -- |
|  |  |  |  |  |
| Self-collection | 100% | 16.53 | 25.24722 | Dom |
| Provider-collection | 100% | 17.36 | 25.26340 | 80 |
|  |  |  |  |  |
| Self-collection | 100% | 16.53 | 25.24722 | Dom |
| Provider-collection | 90% | 16.88 | 25.25740 | 80 |
|  |  |  |  |  |
| Provider-collection | 80% | 16.40 | 25.25112 | 80 |
| Self-collection | 100% | 16.53 | 25.24722 | Dom |
|  |  |  |  |  |
| Provider-collection | 70% | 15.91 | 25.24499 | 80 |
| Self-collection | 100% | 16.53 | 25.24722 | 280 |
|  |  |  |  |  |
| Self-collection | 75% | 15.65 | 25.23595 | Dom |
| Provider-collection | 75% | 16.16 | 25.24806 | 80 |
|  |  |  |  |  |
| Self-collection | 75% | 15.65 | 25.23595 | Dom |
| Provider-collection | 70% | 15.91 | 25.24499 | 80 |
|  |  |  |  |  |
| Provider-collection | 60% | 15.42 | 25.23877 | 80 |
| Self-collection | 75% | 15.65 | 25.23595 | Dom |
|  |  |  |  |  |
| Provider-collection | 50% | 14.92 | 25.23273 | 80 |
| Self-collection | 75% | 15.65 | 25.23595 | 230 |
|  |  |  |  |  |
| Self-collection | 50% | 14.78 | 25.22467 | Dom |
| Provider-collection | 50% | 14.92 | 25.23273 | 80 |
|  |  |  |  |  |
| Provider-collection | 40% | 14.42 | 25.22467 | 80 |
| Self-collection | 50% | 14.78 | 25.22683 | Dom |
|  |  |  |  |  |
| Provider-collection | 30% | 13.92 | 25.22095 | 80 |
| Self-collection | 50% | 14.78 | 25.22467 | 230 |

^a^ Dom: dominated strategy ( i.e., those that are more costly and less effective or have higher ICERs than more effective options); ICER: incremental cost-effectiveness ratio; I$: 2011 international dollars; YLS: year of life saved. Uganda per capita GDP: I$1,690.

^b^ ICERs are provided for each pair of self-collection and provider-collection population screening coverage levels; within each pair, strategies are listed in order of increasing cost. We assume achievable coverage with self-collection is equivalent or higher than achievable population coverage with provider-collection. Natural history refers to a scenario with no screening.

^c^ Discounted lifetime cost per woman and discounted life expectancy represent the mean across 50 input parameter sets.

**Appendix** **Table 12. Sensitivity analysis: District size based on 25th percentile of Ugandan districts for per-woman allocation of programmatic costs (base case: 50th percentile). Incremental cost-effectiveness ratios for clinic-based provider-collection versus a self-collection campaign, by population screening coverage level.^a^**

| **Strategy**^b^ | **Population screening coverage**^b^ | **Discounted lifetime cost per woman**^c^ | **Discounted life expectancy, mean**^c^ | **ICER (I$/YLS), mean**^c^ |
| --- | --- | --- | --- | --- |
| Natural history | 0% | 12.44 | 25.20253 | -- |
|  |  |  |  |  |
| Self-collection | 100% | 16.52 | 25.25676 | 80 |
| Provider-collection | 100% | 17.36 | 25.26340 | 130 |
|  |  |  |  |  |
| Self-collection | 100% | 16.52 | 25.25676 | 80 |
| Provider-collection | 90% | 16.88 | 25.25740 | 570 |
|  |  |  |  |  |
| Provider-collection | 80% | 16.40 | 25.25112 | Dom |
| Self-collection | 100% | 16.52 | 25.25676 | 80 |
|  |  |  |  |  |
| Provider-collection | 70% | 15.91 | 25.24499 | Dom |
| Self-collection | 100% | 16.52 | 25.25676 | 80 |
|  |  |  |  |  |
| Self-collection | 75% | 15.66 | 25.24316 | 80 |
| Provider-collection | 75% | 16.16 | 25.24806 | 100 |
|  |  |  |  |  |
| Self-collection | 75% | 15.66 | 25.24316 | 80 |
| Provider-collection | 70% | 15.91 | 25.24499 | 140 |
|  |  |  |  |  |
| Provider-collection | 60% | 15.42 | 25.23878 | Dom |
| Self-collection | 75% | 15.66 | 25.24316 | 80 |
|  |  |  |  |  |
| Provider-collection | 50% | 14.92 | 25.23273 | Dom |
| Self-collection | 75% | 15.66 | 25.24316 | 80 |
|  |  |  |  |  |
| Self-collection | 50% | 14.81 | 25.22942 | Dom |
| Provider-collection | 50% | 14.92 | 25.23273 | 80 |
|  |  |  |  |  |
| Provider-collection | 40% | 14.42 | 25.22683 | 80 |
| Self-collection | 50% | 14.81 | 25.22943 | 150 |
|  |  |  |  |  |
| Provider-collection | 30% | 13.92 | 25.22095 | 80 |
| Self-collection | 50% | 14.81 | 25.22943 | 110 |

^a^ Dom: dominated strategy ( i.e., those that are more costly and less effective or have higher ICERs than more effective options); ICER: incremental cost-effectiveness ratio; I$: 2011 international dollars; YLS: year of life saved. Uganda per capita GDP: I$1,690.

^b^ ICERs are provided for each pair of self-collection and provider-collection population screening coverage levels; within each pair, strategies are listed in order of increasing cost. We assume achievable coverage with self-collection is equivalent or higher than achievable population coverage with provider-collection. Natural history refers to a scenario with no screening.

^c^ Discounted lifetime cost per woman and discounted life expectancy represent the mean across 50 input parameter sets.

**Appendix** **Table 13. Sensitivity analysis: District size based on 75th percentile of Ugandan districts for per-woman allocation of programmatic costs (base case: 50th percentile). Incremental cost-effectiveness ratios for clinic-based provider-collection versus a self-collection campaign, by population screening coverage level.^a^**

| **Strategy**^b^ | **Population screening coverage**^b^ | **Discounted lifetime cost per woman**^c^ | **Discounted life expectancy, mean**^c^ | **ICER (I$/YLS), mean**^c^ |
| --- | --- | --- | --- | --- |
| Natural history | 0% | 12.44 | 25.20253 | -- |
|  |  |  |  |  |
| Self-collection | 100% | 16.39 | 25.25676 | 70 |
| Provider-collection | 100% | 17.36 | 25.26340 | 150 |
|  |  |  |  |  |
| Self-collection | 100% | 16.39 | 25.25676 | 70 |
| Provider-collection | 90% | 16.88 | 25.25740 | 760 |
|  |  |  |  |  |
| Self-collection | 100% | 16.39 | 25.25676 | 70 |
| Provider-collection | 80% | 16.40 | 25.25112 | Dom |
|  |  |  |  |  |
| Self-collection | 75% | 15.53 | 25.24316 | 80 |
| Provider-collection | 75% | 16.16 | 25.24806 | 130 |
|  |  |  |  |  |
| Self-collection | 75% | 15.53 | 25.24316 | 80 |
| Provider-collection | 70% | 15.91 | 25.24499 | 210 |
|  |  |  |  |  |
| Provider-collection | 60% | 15.42 | 25.23877 | Dom |
| Self-collection | 75% | 15.53 | 25.24316 | 80 |
|  |  |  |  |  |
| Self-collection | 50% | 14.68 | 25.22943 | Dom |
| Provider-collection | 50% | 14.92 | 25.23273 | 80 |
|  |  |  |  |  |
| Provider-collection | 40% | 14.42 | 25.22683 | 80 |
| Self-collection | 50% | 14.68 | 25.22943 | 100 |
|  |  |  |  |  |
| Provider-collection | 30% | 13.92 | 25.22095 | 80 |
| Self-collection | 50% | 14.68 | 25.22943 | 90 |

^a^ Dom: dominated strategy ( i.e., those that are more costly and less effective or have higher ICERs than more effective options); ICER: incremental cost-effectiveness ratio; I$: 2011 international dollars; YLS: year of life saved. Uganda per capita GDP: I$1,690.

^b^ ICERs are provided for each pair of self-collection and provider-collection population screening coverage levels; within each pair, strategies are listed in order of increasing cost. We assume achievable coverage with self-collection is equivalent or higher than achievable population coverage with provider-collection. Natural history refers to a scenario with no screening.

^c^ Discounted lifetime cost per woman and discounted life expectancy represent the mean across 50 input parameter sets.

**Appendix** **Table 14. Sensitivity analysis: Test sensitivity/specificity on self-collected specimens is equivalent to provider-collected specimens (0.89/0.82) (base case: 0.77, 0.82). Incremental cost-effectiveness ratios for clinic-based provider-collection versus a self-collection campaign, by population screening coverage level.^a^**

| **Strategy**^b^ | **Population screening coverage**^b^ | **Discounted lifetime cost per woman**^c^ | **Discounted life expectancy, mean**^c^ | **ICER (I$/YLS), mean**^c^ |
| --- | --- | --- | --- | --- |
| Natural history | 0% | 12.41 | 25.20221 | -- |
|  |  |  |  |  |
| Self-collection | 100% | 16.20 | 25.26340 | 60 |
| Provider-collection | 100% | 17.36 | 25.26340 | Dom |
|  |  |  |  |  |
| Self-collection | 100% | 16.20 | 25.26340 | 60 |
| Provider-collection | 90% | 16.88 | 25.25740 | Dom |
|  |  |  |  |  |
| Self-collection | 100% | 16.20 | 25.26340 | 60 |
| Provider-collection | 80% | 16.40 | 25.25112 | Dom |
|  |  |  |  |  |
| Self-collection | 75% | 15.41 | 25.24806 | 70 |
| Provider-collection | 75% | 16.16 | 25.24806 | Dom |
|  |  |  |  |  |
| Self-collection | 75% | 15.41 | 25.24806 | 70 |
| Provider-collection | 70% | 15.91 | 25.24499 | Dom |
|  |  |  |  |  |
| Self-collection | 75% | 15.41 | 25.24806 | 70 |
| Provider-collection | 60% | 15.42 | 25.23878 | Dom |
|  |  |  |  |  |
| Self-collection | 50% | 14.62 | 25.23273 | 70 |
| Provider-collection | 50% | 14.92 | 25.23273 | Dom |
|  |  |  |  |  |
| Provider-collection | 40% | 14.42 | 25.22683 | Dom |
| Self-collection | 50% | 14.62 | 25.23273 | 70 |
|  |  |  |  |  |
| Provider-collection | 30% | 13.92 | 25.22095 | Dom |
| Self-collection | 50% | 14.62 | 25.23273 | 70 |

^a^ Dom: dominated strategy ( i.e., those that are more costly and less effective or have higher ICERs than more effective options); ICER: incremental cost-effectiveness ratio; I$: 2011 international dollars; YLS: year of life saved. Uganda per capita GDP: I$1,690.

^b^ ICERs are provided for each pair of self-collection and provider-collection population screening coverage levels; within each pair, strategies are listed in order of increasing cost. We assume achievable coverage with self-collection is equivalent or higher than achievable population coverage with provider-collection. Natural history refers to a scenario with no screening.

^c^ Discounted lifetime cost per woman and discounted life expectancy represent the mean across 50 input parameter sets.

**Appendix** **Table 15. Sensitivity analysis: Programmatic costs for provider-collection equivalent to self-collection campaign (base case: I$0 per woman screened). Incremental cost-effectiveness ratios for clinic-based provider-collection versus a self-collection campaign, by population screening coverage level.^a^**

| **Strategy**^b^ | **Population screening coverage**^b^ | **Discounted lifetime cost per woman**^c^ | **Discounted life expectancy, mean**^c^ | **ICER (I$/YLS), mean**^c^ |
| --- | --- | --- | --- | --- |
| Natural history | 0% | 12.41 | 25.20221 | -- |
|  |  |  |  |  |
| Self-collection | 100% | 16.45 | 25.25676 | 70 |
| Provider-collection | 100% | 17.56 | 25.26340 | 170 |
|  |  |  |  |  |
| Self-collection | 100% | 16.45 | 25.25676 | 70 |
| Provider-collection | 90% | 17.08 | 25.25740 | 990 |
|  |  |  |  |  |
| Self-collection | 100% | 16.45 | 25.25676 | 70 |
| Provider-collection | 80% | 16.61 | 25.25112 | Dom |
|  |  |  |  |  |
| Self-collection | 75% | 15.59 | 25.24316 | 80 |
| Provider-collection | 75% | 16.37 | 25.24806 | 160 |
|  |  |  |  |  |
| Self-collection | 75% | 15.59 | 25.24316 | 80 |
| Provider-collection | 70% | 16.11 | 25.24499 | 280 |
|  |  |  |  |  |
| Self-collection | 75% | 15.59 | 25.24316 | 80 |
| Provider-collection | 60% | 15.63 | 25.23877 | Dom |
|  |  |  |  |  |
| Self-collection | 50% | 14.75 | 25.22943 | 90 |
| Provider-collection | 50% | 15.13 | 25.23273 | 120 |
|  |  |  |  |  |
| Provider-collection | 40% | 14.63 | 25.22683 | Dom |
| Self-collection | 50% | 14.75 | 25.22942 | 90 |
|  |  |  |  |  |
| Provider-collection | 30% | 14.12 | 25.22095 | Dom |
| Self-collection | 50% | 14.75 | 25.22943 | 90 |

^a^ Dom: dominated strategy ( i.e., those that are more costly and less effective or have higher ICERs than more effective options); ICER: incremental cost-effectiveness ratio; I$: 2011 international dollars; YLS: year of life saved. Programmatic costs of provider-collection were assumed to be equivalent to programmatic costs of a self-collection campaign at the comparable coverage level (i.e., I$0.52 at 100% coverage, I$0.58 at 90% coverage, I$0.65 at 80% coverage, I$0.70 at 75% coverage, I$0.74 at 70% coverage, I$0.86 at 60% coverage, I$1.04 at 50% coverage, I$1.30 at 40% coverage, and I$1.73 at 30% coverage). Uganda per capita GDP: I$1,690.

^b^ ICERs are provided for each pair of self-collection and provider-collection population screening coverage levels; within each pair, strategies are listed in order of increasing cost. We assume achievable coverage with self-collection is equivalent or higher than achievable population coverage with provider-collection. Natural history refers to a scenario with no screening.

^c^ Discounted lifetime cost per woman and discounted life expectancy represent the mean across 50 input parameter sets.

**Appendix Figure 1.** Selected model output from the top 50 input parameter sets compared with empirical data (i.e., calibration targets) on age-specific prevalence of high-risk HPV in Uganda, based on a relative light unit cut-off value of 0.5 in the START-UP study [[2](#_ENREF_2)]. Bold lines represent the 95% confidence intervals around the empirical data, and gray circles represent model output from each of the top 50 input parameter sets. Due to the small number of women aged 55 to 60 years in the START-UP study, the calibration target in this age group is highly uncertain.

**Appendix Figure 2.** Selected model output from the top 50 input parameter sets compared with empirical data (i.e., calibration targets) on age-specific cancer incidence in Uganda (Kyadondo registry, 2003-2007)[[13](#_ENREF_13)]. Bold lines represent the 95% confidence intervals around the empirical data, and gray circles represent model output from each of the top 50 input parameter sets.

**Appendix Figure 3.** Reduction in lifetime risk of cervical cancer for clinic-based provider-collection (blue bars) versus a self-collection campaign (red bars) (base case analysis) is displayed on the y-axis as screening coverage level is varied along the x-axis.

**References**

1. Moses E, Pedersen HN, Mitchell SM, Sekikubo M, Mwesigwa D, Singer J, et al. Uptake of community-based, self-collected HPV testing vs. visual inspection with acetic acid for cervical cancer screening in Kampala, Uganda: preliminary results of a randomised controlled trial. Trop Med Int Health 2015; 20: 1355-1367.

2. Jeronimo J, Bansil P, Lim J, Peck R, Paul P, Amador JJ, et al. A multicountry evaluation of careHPV testing, visual inspection with acetic acid, and papanicolaou testing for the detection of cervical cancer. Int J Gynecol Cancer 2014; 24: 576-585.

3. United Nations DoEaSA, Population Division (2013) World Population Prospects, The 2012 Revision. Geneva.

4. Organization. WH Global Health Observatory Data Repository.

5. Geohive (2015).

6. (2013) World Development Indicators. World Bank.

7. Programme UND (2014) International Human Development Indicators.

8. Campos NGM, M.; Alfaro, K.; Gage, J.C.; Castle, P.E.; Felix, J.; Cremer, M.L.; Kim, J.J. The Comparative and Cost-Effectiveness of HPV-Based Cervical Cancer Screening in El Salvador.

9. Goldie SJ, Gaffikin L, Goldhaber-Fiebert JD, Gordillo-Tobar A, Levin C, Mahe C, et al. Cost-effectiveness of cervical-cancer screening in five developing countries. N Engl J Med 2005; 353: 2158-2168.

10. Campos NG, Tsu, V., Jeronimo, J., Mvundura, M., Lee, K., Kim, J.J. When and how often to screen for cervical cancer in three low- and middle-income countries: A cost-effectiveness analysis. Papillomavirus Research 2015.

11. Campos NG, Kim JJ, Castle PE, Ortendahl JD, O'Shea M, Diaz M, et al. Health and economic impact of HPV 16/18 vaccination and cervical cancer screening in Eastern Africa. Int J Cancer 2012; 130: 2672-2684.

12. Mvundura M, Tsu V Estimating the costs of cervical cancer screening in high-burden Sub-Saharan African countries. Int J Gynaecol Obstet 2014; 126: 151-155.

13. Forman D, Bray, F., Brewster, D.H., Gombe Mbalawa, C., Kohler, B., Piñeros, M., Steliarova-Foucher, E., Swaminathan, R., Ferlay, J. (eds) (2013) Cancer Incidence in Five Continents, Vol. X. Lyon, France: IARC.
